# Different cucumber *CsYUC* genes regulate response to abiotic stresses and flower development

**DOI:** 10.1038/srep20760

**Published:** 2016-02-09

**Authors:** Shuangshuang Yan, Gen Che, Lian Ding, Zijing Chen, Xiaofeng Liu, Hongyin Wang, Wensheng Zhao, Kang Ning, Jianyu Zhao, Kiflom Tesfamichael, Qian Wang, Xiaolan Zhang

**Affiliations:** 1 Department of Vegetable Sciences, Beijing Key Laboratory of Growth and Developmental Regulation for Protected Vegetable Crops, China Agricultural University, Beijing 100193, China

## Abstract

The phytohormone auxin is essential for plant growth and development, and YUCCA (YUC) proteins catalyze a rate-limiting step for endogenous auxin biosynthesis. Despite *YUC* family genes have been isolated from several species, systematic expression analyses of *YUCs* in response to abiotic stress are lacking, and little is known about the function of YUC homologs in agricultural crops. Cucumber (*Cucumis sativus* L.) is a world cultivated vegetable crop with great economical and nutritional value. In this study, we isolated 10 *YUC* family genes (*CsYUCs)* from cucumber and explored their expression pattern under four types of stress treatments. Our data showed that *CsYUC8* and *CsYUC9* were specifically upregulated to elevate the auxin level under high temperature. *CsYUC10b* was dramatically increased but *CsYUC4* was repressed in response to low temperature. *CsYUC10a* and *CsYUC11* act against the upregulation of *CsYUC10b* under salinity stress, suggesting that distinct YUC members participate in different stress response, and may even antagonize each other to maintain the proper auxin levels in cucumber. Further, *CsYUC11* was specifically expressed in the male flower in cucumber, and enhanced tolerance to salinity stress and regulated pedicel and stamen development through auxin biosynthesis in *Arabidopsis*.

The phytohormone auxin plays fundamental roles in plant growth and development, such as shoot and root formation^1^, apical dominance^2^, plant architecture^3^, floral development^4^, gynoecium and fruit development^5^,^6^ and various tropic responses^7^. These biological functions of auxin result from the coordinated actions of auxin biosynthesis, auxin transport and auxin signalling. Auxin biosynthesis was found to take place in tissues such as cotyledons, leaves, flowers, and shoot apex^8^. Synthesis of auxin can be divided into one tryptophan (Trp)-independent and four Trp-dependent pathways^9^,^10^. The Trp-dependent pathways are much well understood than the Trp-independent pathway^10^,^11^. The primary Trp-dependent pathway for auxin biosynthesis is a two-step process, in which Trp was first converted into indole-3-pyruvate (IPA) by TRYPTOPHAN AMINOTRANSFERASE OF ARABIDOPSIS1/TRYPTOPHAN AMINOTRANSFERASE RELATED (TAA1/TAR), and then YUCCA (YUC) flavin-containing monooxygenases catalyzed IPA into indole-3-acetic acid (IAA), the major endogenous auxin^12^,^13^. The second step catalyzed by YUCs is a rate-limiting step for auxin biosynthesis which plays a significant role in most auxin-mediated developmental processes^14^,^15^. This Trp-dependent pathway is highly conserved and widely exists throughout the plant kingdom^11^.

YUC proteins belong to the flavin-containing monooxygenases (FMOs) which had several conserved domains^11^,^16^. The FAD-binding motif and NADPH-binding motif containing the GXGXXG residues were highly conserved across all of the FMOs^16^,^17^, with the FAD-binding motif locating near the N-terminus and the NADPH-binding motif locating in the middle of YUC proteins^18^. The FMO-identifying sequence with FXGXXXHXXXY residues contributes to NADPH binding, and was found in all plant FMOs as well^17^,^18^. *YUC* genes have been identified in many plant species. For example, 11 *YUCs* were identified in *Arabidopsis*, in which *YUC1-6*, *YUC10* and *YUC11* have been confirmed to be involved in local auxin biosynthesis^13^,^15^,^19^,^20^. In addition, one *YUC* gene from petunia^21^, six *YUC* genes from tomato^22^, seven *YUC*-like genes from rice^23^ and eight *YUC* gens from strawberry^24^ have been isolated.

A wealth of evidence indicated that *YUC* genes play important roles in multitude of developmental processes. Loss-of-function of *YUC* family genes resulted in dramatic developmental defects, such as *yuc1 yuc4* mutant produced fewer flowers, *yuc1 yuc4 yuc10 yuc11* mutant displayed one cotyledon with reduced vascular system, and *yuc2 yuc6* plant showed defective stamen development in *Arabidopsis*^15^,^25^,^26^,^27^. Similarly, mutation in *SPARSE INFLORESCENCE1 (SPI1)*, the *YUC1* ortholog in maize, led to reduced number of branches and spikelets^28^. Overexpression of *YUC* genes caused phenotypes mimicking auxin overproduction such as the long hypocotyl phenotype in *Arabidopsis*^14^, leaf aberration and abnormal flower architecture in petunia^21^, and the changes of leaf shape, shoot and root elongation in rice^23^,^29^.

In addition to developmental regulation, *YUC* family genes are also involved in plant response to abiotic stresses. High temperature led to hypocotyl elongation and increased IAA level in *Arabidopsis*^30^. *PHYTOCHROME-INTERACTING FACTOR* 4 (*PIF4*), which encodes a basic helix-loop-helix (bHLH) transcription factor, regulates high temperature-induced hypocotyl elongation through up-regulation of *YUC8* in *Arabidopsis*^31^. On the other hand, high temperature resulted in male sterility in the developing anthers of barley and *Arabidopsis*, which was caused by reduced auxin level and repressed expression of *YUC2* and *YUC6*^32^. The activation-tagged mutant *yuc-1D* in *Arabidopsis* showed enhanced resistant to water deficit^33^. Similarly, overexpression of *AtYUC6* increased resistance to drought stress in potato^34^, whereas inactivation of *CONSTITUTIVELY WILTED 1* (rice ortholog to *YUCs*) impaired the resistant to water deficit in rice^35^. In *Arabidopsis*, auxin concentrations were remarkably elevated under shade condition, and the expression of *YUC2*, *YUC5*, *YUC8* and *YUC9* were up-regulated as expected^36^.

However, systematic expression analyses of YUC family genes in response to abiotic stress are lacking, and little is known about the function of YUC homologs in agricultural crops. Cucumber (*Cucumis sativus* L.) is an important vegetable crop with great economical and nutritional value. In this study, we isolated 10 *YUC* family genes in cucumber, and explored the expression of all 10 *CsYUCs* in response to high temperature, low temperature, salinity stress and shading treatment. Our data showed that distinct *CsYUC* members mediated different abiotic stresses. Further, *CsYUC11* was specifically expressed in male flower in cucumber, and enhanced tolerance to salinity stress and regulated pedicel and stamen development in *Arabidopsis*.

## Results

### Identification of *CsYUCs* in cucumber and gene structure analysis

**Figure 1:**
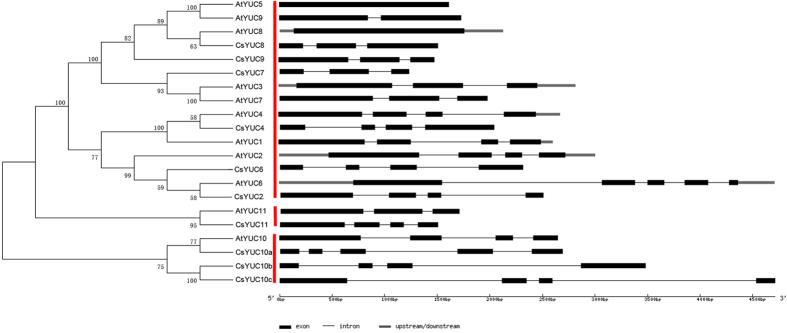
Phylogenetic analysis and gene structure of *YUC* family genes in cucumber and *Arabidopsis*. The unrooted tree including 11 *Arabidopsis* YUCs and 10 cucumber YUC genes was constructed by MEGA5 software using the Neighbor-joining (NJ) method. Gene structure was generated in http://gsds.cbi.pku.edu.cn/. Black boxes and lines represent exons and introns, respectively. Upstream and downstream sequences are indicted by gray boxes.

To identify the YUC family members from cucumber, we performed BLAST search in the Cucumber Genome DataBase^37^ based on the amino acid sequence information of the 11 *Arabidopsis* YUC genes. 10 CsYUCs were identified and named accordingly based on the best hit in *Arabidopsis* (Figure S1, Supplemental Table S1). There are no homologs for AtYUC1, AtYUC3 or AtYUC5 in cucumber, but instead, there are three homologs for AtYUC10, which are named as CsYUC10a, CsYUC10b, and CsYUC10c, with the highest similarity in CsYUC10a (56.44%) and least similarity in CsYUC10c (44.16%) (Supplemental Table S1). Protein similarity varies from 43.52% (AtYUC7/CsYUC7) to 78.87% (AtYUC8/CsYUC8) between YUC families in cucumber and *Arabidopsis* (Supplemental Figure S1). Phylogenetic analysis using the full-length protein sequence of AtYUCs and CsYUCs showed that all the YUCs can be divided into three clades (red lines in Fig. 1). The first clade is the biggest, consisting of 6 CsYUCs and 9 AtYUCs. The second clade is made exclusively of CsYUC11 and AtYUC11, while AtYUC10 and CsYUC10s constitute the third clade.

Gene structure analysis was performed to compare the exon-intron organization of *CsYUCs* and *AtYUCs* (Fig. 1). *CsYUC* genes contained three to five exons, in which *CsYUC2*, *CsYUC4*, *CsYUC7*, *CsYUC10b*, *CsYUC10c* have the same gene structure as those in *Arabidopsis*, *CsYUC6* has one exon less than *AtYUC6*, while the remaining four *CsYUCs* including *CsYUC8*, *CsYUC9*, *CsYUC10a* and *CsYUC11* contain one or two more exons than the corresponding *AtYUCs* (Fig. 1). To further compare the gene structure between *CsYUCs* and their *Arabidopsis* homologs, each exon was comparatively analyzed in detail (Supplemental Table S2). For each gene, the first exon is generally the longest, and the third exon is the shortest (Supplemental Table S2). Despite exon numbers are variable in the *YUC* families, the full length coding sequences are quite similar (around 1200bp) except for *CsYUC7* (Supplemental Table S2). The ten *CsYUC* genes are unevenly distributed on five chromosomes, with *CsYUC7*, *CsYUC9*, *CsYUC10a* located on chromosome 3, while no *CsYUC* gene is mapped to chromosome 4 or chromosome 5 (Supplemental Figure S2), the accession number and the exact genomic position of each *CsYUC* gene were provided in Supplemental Table S3.

**Figure 2:**
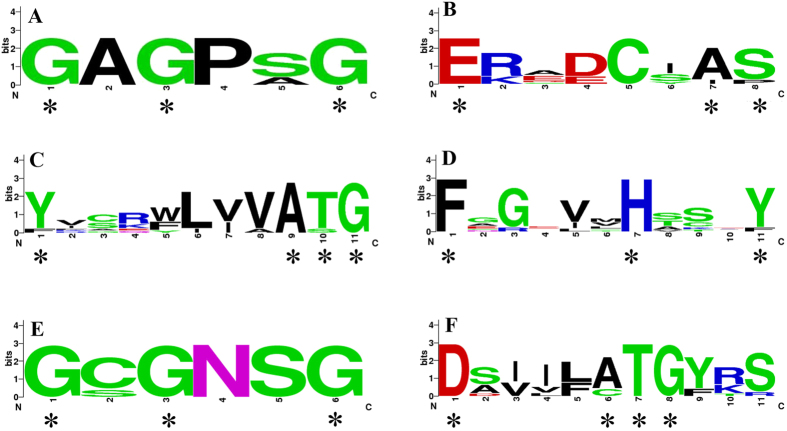
The conserved flavin monooxygenase (FMO) domains in the CsYUCs. (**A–F**) The six relatively conserved FMO domains across all CsYUC proteins. The FAD-binding domain (**A**), GC-motif (**B**), ATG-Containing motif 1 (**C**), FMO-identifying sequence (**D**), NADPH domain (**E**), ATG-Containing motif 2 (**F**). The sequence logos of the 6 motifs are based on full-length alignments of the 10 CsYUC proteins. The overall height of each stack represents the conservation of the sequence at that position, and the height of letters within each stack indicates the relative frequency of the respective amino acid. The asterisk shows the position of the conserved amino acid that is identical cross all FMOs proteins.

Given that plant YUC-like flavin monooxygenases (FMOs) have several conserved motifs that play important roles in many aspects of plant development^16^,^18^, the full length of the ten CsYUCs protein sequences were aligned and the sequence logo for each FMO domain was produced to explore how conserved of these motifs (Fig. 2, Supplemental Figure S3). From N-terminus to C-terminus, six conserved FMO domains were identified, which are the FAD-binding motif, GC motif, ATG-containing motif 1, FMO-identifying sequence, NADPH-binding motif and ATG-containing motif 2 (Supplemental Figure S3). Previous studies showed that the GC-motif help stabilization of the FAD-binding, ATG-containing motif 1 plays a bridge role between FAD-binding motif and NADPH-binding motif, FMO-identifying sequence contributes to NADPH binding, while the ATG-containing motif 2 provides linkage between NADPH site and the active site^24^. Among all FMOs in the CsYUCs, the FAD-binding motif at the N-terminus and the NADPH-binding motif in the middle are the most conserved and display consensus core amino acids (asterisks in Fig 2A,E, Supplemental Figure S3). Distinct from other YUC members in cucumber, CsYUC6 and CsYUC7 lack of FAD-binding motif and GC motif (Supplemental Figure S3). CsYUC10b displays variations in the highly conserved sites of GC motif and FMO-identifying sequence, in which the residues of A and S are replaced by I and P in the GC motif, and the residue of Y is replaced by F in the FMO-identifying sequence, implying that CsYUC10b may play different functions among the cucumber CsYUCs. In the ATG-containing motif 1 and 2, CsYUC11 and CsYUC10s display several variants at the conserved amino acid sites, suggesting that YUCs in the clade 2 and clade 3 may play distinct roles than those in the clade 1 (Fig. 1, Supplemental Figure S3).

### Expression patterns of *CsYUCs* in cucumber

**Figure 3:**
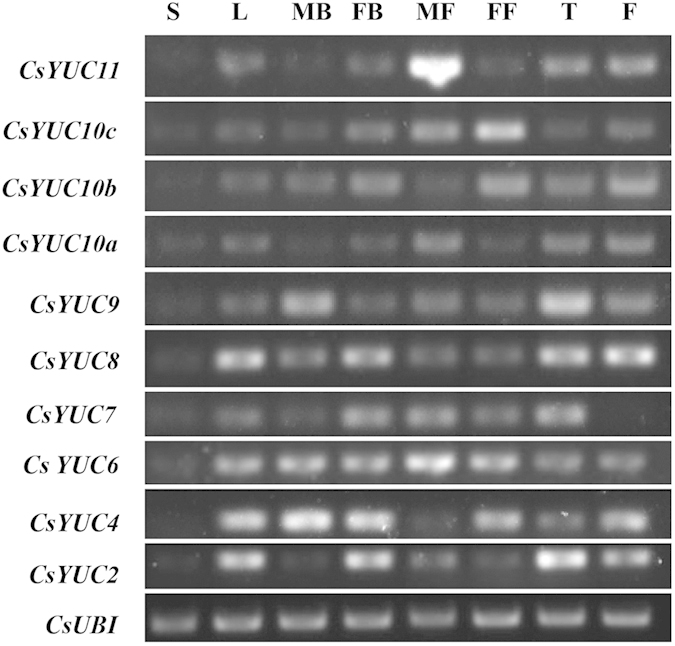
Semi-quantitative RT-PCR analysis of *CsYUCs* in cucumber. Transcripts of *CsYUCs* were analyzed in stem (S), leaves (L), male floral buds (MB), female floral buds (FB), opened male flowers (MF), opened female flowers (FF), tendrils (T) and fruits (F). The cucumber *UBIQUITIN* gene was used as an internal control to normalize the expression data.

To explore the expression patterns of *CsYUCs*, semi-quantitative RT-PCR was used to examine the transcript levels in different tissues including stem (S), leaves (L), male floral buds (MB), female floral buds (FB), opened male flowers (MF), opened female flower (FF), tendril (T) and fruit (F) (Fig. 3). In the stem, all *CsYUCs* show no or very low expression, suggesting that stem is not a place for auxin sysnthesis. For all other tissues, most *CsYUCs* display ubiquitous expression, but divergence does exist. For example, *CsYUC4* and *CsYUC10b* display somewhat a complementary expression pattern to *CsYUC11*, in which *CsYUC4* and *CsYUC10b* are highly expressed in all tissues except the opened male flower, whereas *CsYUC11* is specifically enriched in the opened male flower but low in other tissues (Fig. 3). Expression of *CsYUC2* and *CsYUC8* are similarly enriched in leaves, female floral buds, tendrils and fruits, while *CsYUC7* expression is exclusively repressed in the fruits (Fig. 3).

### Distinct *CsYUCs* contribute to auxin production under different abiotic stresses

**Figure 4:**
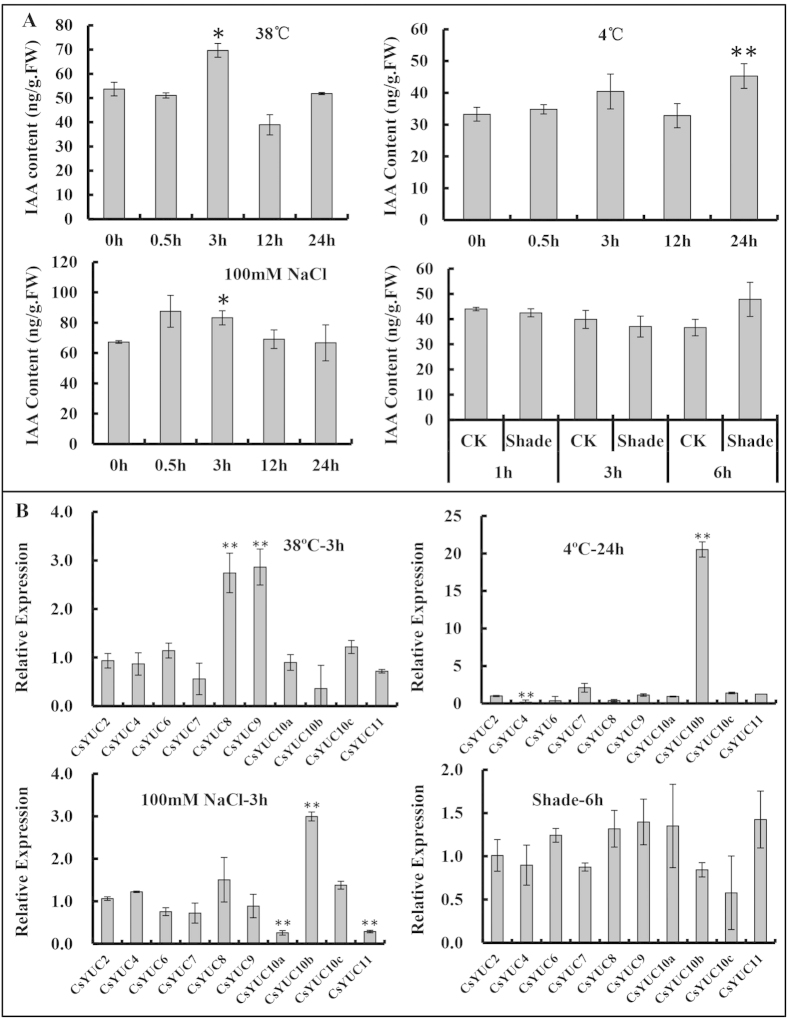
Endogenous IAA level and expression of *CsYUCs* in response to different abiotic stresses. (**A**) IAA content of 21-days-old seedlings under different abiotic stresses. (**B**) Expression of *CsYUCs* in response to abiotic stresses. The bars represent the standard deviation of three biological replicates. Asterisks and double asterisks indicate significant difference between treated and untreated (0 h) control at P < 0.05 and P < 0.01 (unpaired *t*-test), respectively.

Cucumber is an agricultural crop that is very sensitive to abiotic stress, such as high or low temperature, salinity and shading^38^,^39^,^40^. To investigate whether *CsYUCs* mediate the stress responses in cucumber, auxin concentration and *CsYUCs* expression were examined in the cucumber seedlings under high temperature (38 °C), low temperature (4 °C), salinity stress (NaCl 100 mM) and shading treatment. As shown in Fig. 4, IAA abundance accumulated significantly under high temperature for 3 hours, consistent with previous finding that high temperature elevates IAA level in *Arabidopsis*^30^ (Fig. 4A). Given that YUC enzymes play essential roles in the auxin biosynthesis^11^, increased IAA level at high temperature may result from induced *CsYUCs* expression. Therefore, transcript levels of *CsYUCs* were examined in cucumber seedlings under high temperature for 3 h. Among all the 10 *CsYUCs*, *CsYUC8* and *CsYUC9* were significantly induced by high temperature, indicating that *CsYUC8* and *CsYUC9* may specifically responsible for the elevated IAA level in response to high temperature in cucumber (Fig. 4A,B), similar to the finding that high temperature induces *YUC8* expression and increases endogenous IAA level in *Arabidopsis*^31^. Parallel experiments indicated that low temperature and high salinity also elevated free IAA level in cucumber (Fig. 4A), which may mainly due to the dramatically increased *CsYUC10b* expression (Fig. 4B). Interestingly, the expression of *CsYUC10b* was induced 20 fold while *CsYUC4* was decreased 5.5 fold under low temperature (Fig. 4B). Similarly, transcription of *CsYUC10b* was increased but *CsYUC10a* and *CsYUC11* were repressed under salinity stress, consistent with the complementary expression patterns as revealed by semiquantitative RT-PCR (Fig. 3), suggesting that different YUC members may antagonize each other to maintain the proper auxin level in cucumber. The relationship between shading and auxin was also explored, but no obvious changes were observed for either auxin content or expression of *CsYUC* genes in cucumber (Fig. 4A,B).

### *CsYUC11* is specifically enriched in male floral organs

**Figure 5:**
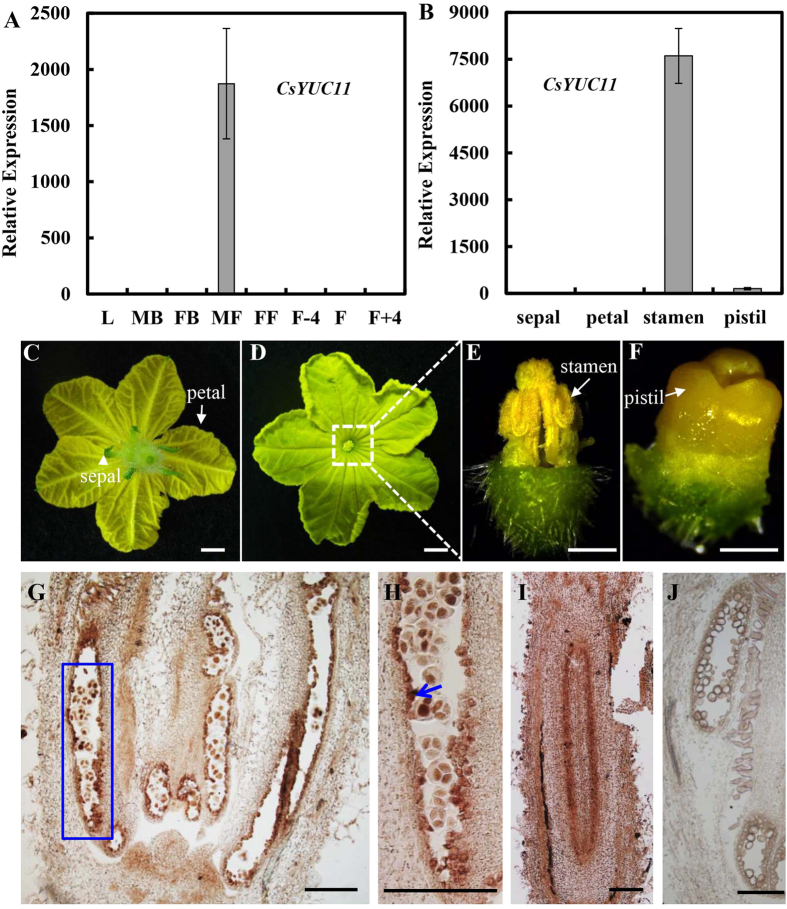
Expression analysis of *CsYUC11* by qRT-PCR and *in situ* hybridization in cucumber. (**A–B**) Quantitative real time RT-PCR analysis of *CsYUC11* in different tissues (**A**) and different floral organs in opened male flowers (**B**). L, leaves; MB, male floral buds; FB, female floral buds; MF, opened male flowers; FF, opened female flowers. F − 4, fruits 4 days before anthesis. F, fruits on anthesis. F + 4, fruits 4 days after anthesis. The cucumber *UBIQUITIN* gene was used as an internal control to normalize the expression. (**C–F**) Opened male flower showing the four floral organs used for qRT-PCR analysis in (**B**). (**C**) Back view of male flower, the arrowhead and arrow indicate sepal and petal, respectively. (**D–F**) Top view of male flower, the stamen (**E**) and the repressed pistil (**F**) are the magnification view of the dotted square in (**D**). (**G–J**) *in situ* hybridization analysis of *CsYUC11* in male and female floral buds in cucumber. (**G,H**) Male floral bud at stage 10 (**G,H**) is the magnification view of the blue rectangle in (**G**). Arrow shows the high expression of *CsYUC11* in tapetum cell layer. (**I**) Female floral bud at stage 8. (**J**) Male floral bud with sense probe. Scale bars represent 1cm in (**C**,**D**), 1 mm in (**E**,**F**), and 200 μm in (**G**–**J**).

The semiquantitative RT-PCR indicated that *CsYUC11* may specifically express in the opened male flowers in cucumber (Fig. 3). To further characterize the expression pattern of *CsYUC11*, quantitative real time PCR and *in situ* hybridization analysis were performed in different tissues of cucumber (Fig. 5). qRT-PCR confirmed that *CsYUC11* is specifically enriched in the opened male flowers (over 1800 fold higher in male flowers than that in the leaves). To identify *CsYUC11* is expressed in which floral organs of male flower, opened male flowers were divided into sepals, petals, stamens and repressed pistils, and qRT-PCR were performed in each type of floral organs (Fig. 5B–F). Our data showed that *CsYUC11* is strongly enriched in the stamens, and weakly expressed in the repressed pistils (Fig. 5B). Next, *in situ* hybridization was used to detect the spatial and temporal distribution of *CsYUC11* (Fig. 5G–J). As expected, *CsYUC11* signal is mainly detected in the tapetum cell layer and the microspores in anthers (Fig. 5G,H). In the female floral bud, *CsYUC11* is weakly expressed in the placenta (Fig. 5I), while no signal was detected upon hybridization with the sense probe of *CsYUC11* (Fig. 5J).

### Ectopic expression of *CsYUC11* in *Arabidopsis*

**Figure 6:**
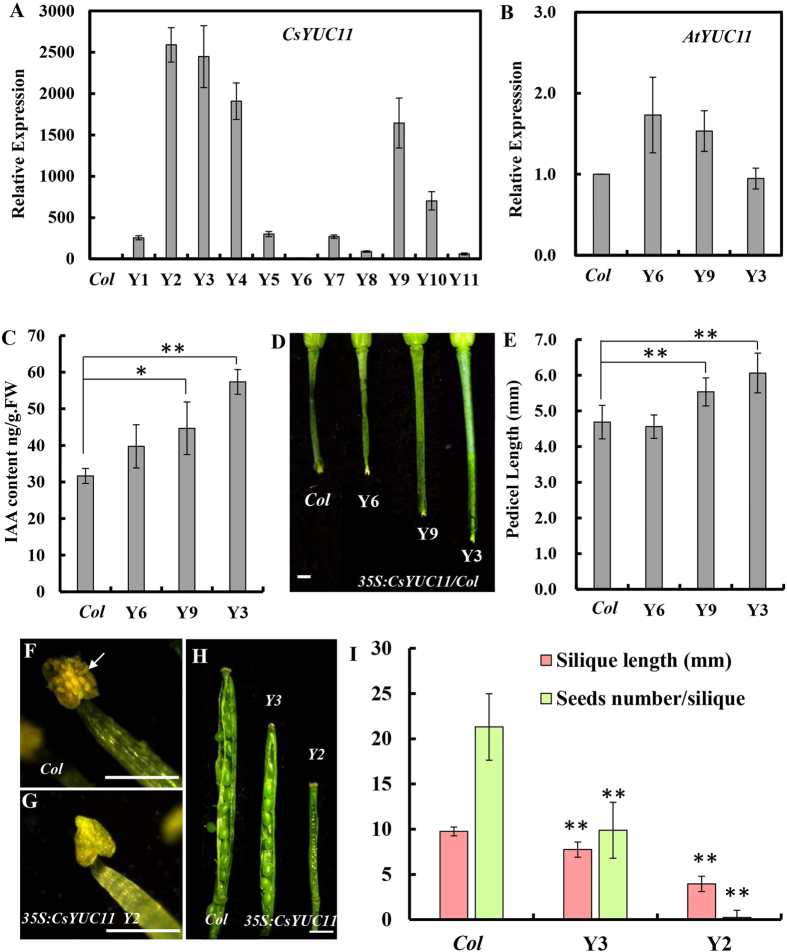
Ectopic expression of *CsYUC11* in *Arabidopsis*. (**A–B**) qRT-PCR analysis of *CsYUC11* (**A**) and *AtYUC11* (**B**) in the inflorescence apex of WT (*Col*) and *35S:CsYUC11* transgenic lines. (**C**) Endogenous IAA content of WT (*Col*) and three transgenic lines. (**D**) The fruit pedicel of *Col* and three transgenic lines at stage 17. (**E**) Quantification of pedicel length in *Col* and transgenic lines (n = 10). (**F–G**) Mature stamens in *Col* (**F**) and *35S:CsYUC11* transgenic line 2 (**G**). The arrow indicates the opened pollen sac. (**H**) The siliques of *Col* and *35S:CsYUC11* line 3 and 2 at the same developmental stage. (**I**) Quantification of silique length and seed number per silique in *Col* and transgenic lines (n = 12). Double asterisks indicate significant difference between *Col* and transgenic lines at P < 0.01 (unpaired t- test). Scale bars represent 1 mm in D and H, and 250 μm in (**F–G**).

To investigate the biological function of *CsYUC11*, we ectopically expressed the *CsYUC11* coding sequence under the 35S promoter of Cauliflower mosaic virus (CaMV) in *Arabidopsis* wild-type *Columbia* (*Col*), and 11 independent transgenic lines were obtained (Fig. 6A). The phenotypes of the transgenic lines vary from mild to medium to severe, which are in consistent with the respective expression level. Thus we chose three representive lines Y6, Y9 and Y3 for further characterization. To explore whether ectopic expression of *CsYUC11* affect the transcription of *AtYUC11* in *Arabidopsis*, we checked the expression of *AtYUC11* in the transgenic lines. As shown in Fig. 6B, there is no obvious difference in the expression of *AtYUC11*, suggesting that the phenotypes of 35S:CsYUC11 lines were due to the function of CsYUC11, not AtYUC11. As the expression level of *CsYUC11* increase, the pedicel length as well as the IAA content increase progressively (Fig. 6C,D). The mean pedicel length of Y3 was up to 6.1 ± 0.6 mm while that of *Col* was only 4.7 ± 0.5 mm (Fig. 6E), implying that *CsYUC11* promotes pedicel elongation through auxin biosynthesis in *Arabidopsis*. Given that *CsYUC11* is highly expressed in stamens, we examined the stamen development in the transgenic lines. Despite the number of stamens remain unchanged, the pollens are defective and the anthers fail to open at stage 13 in the severe transgenic lines, a prerequisite for proper pollination and seed set in *Arabidopsis* (Fig. 6F,G)^41^. Consequently, the silique length and the seed number per silique were significantly reduced in the transgenic lines (Fig. 6H,I). In the most severe line Y2, only 3 seeds were obtained in the 12 siliques we examined (Fig. 6I, Supplemental Figure S4). Therefore, our data suggested that *CsYUC11* regulates silique elongation and seed production through the essential role in stamen development in *Arabidopsis.*

**Figure 7:**
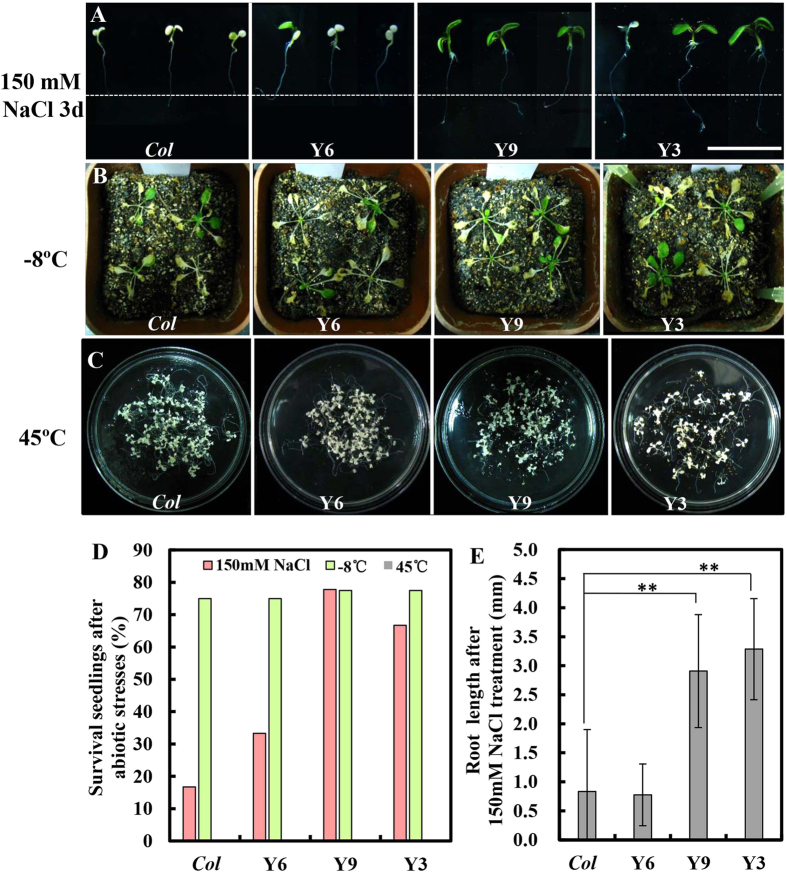
*CsYUC11* mediates abiotic stress in *Arabidopsis*. (**A**) Root elongation after 150 mM NaCl treatment (**E**). (**B**) Tolerance of 3-week-old seedlings at −8 °C for 2 hours. The pictures were taken 7 days after treatments. (**C**) Tolerance of seedlings after high temperature treatment (45 °C for 2.5 h). (**D**) Quantification of the survival rate under abiotic stress treatment. Double asterisks indicate significant difference between *Col* and transgenic lines at P < 0.01 (unpaired t- test). Scale bars represent 1 cm in (**A**).

To further characterize the function of CsYUC11 under abiotic stress, 35S:CsYUC11 transgenic *Arabidopsis* plants were subjected to salinity, freezing and heat stress treatments using the homozygous lines Y6, Y9 and Y3. Compared to the WT, the 35S:CsYUC11 transgenic seedlings displayed reduced suppression of root elongation upon 150 mM NaCl stress for 3 d (Fig. 7A,E), and thus a higher survival rate (Fig. 7D), suggesting that *CsYUC11* enhances plant tolerance to salinity stress, which is consistent with the expression data in which salinity stress significantly repressed the transcription of *CsYUC11* in cucumber (Fig. 4B). Under freezing (−8 °C) or heat (45 °C) treatments, there is no obvious difference between WT and transgenic plants (Fig. 7B–D), consistent with the fact that expression of *CsYUC11* is not respond to freezing or heat stress (Fig. 4B).

## Discussion

### Structural conservation and divergence of cucumber *CsYUCs*

The step converting IPA into IAA catalyzed by YUCs is a rate-limiting step for auxin biosynthesis^12^,^13^, and thus YUC enzymes were widely distributed and characterized in higher plants including *Arabidopsis*, rice, tomato, petunia and strawberry^21^,^22^,^23^,^24^. In this study, we isolated 10 CsYUC family genes in cucumber (Figure S1, Supplemental Table S1). Phylogenetic and gene structure analysis showed that the 10 CsYUCs can be divided into three clades, with most CsYUCs located in the first clade (Fig. 1), similar to those in *Arabidopsis,* rice and strawberry^15^,^24^. *CsYUCs* were unevenly distributed on five chromosomes (Supplemental Figure S2), and all *CsYUCs* have similar length of coding sequence (around 1200bp) except for *CsYUC7* (Supplemental Table S2). At the protein sequence level, cucumber CsYUCs displayed six conserved motifs (Fig. 2 and Supplemental Table S3). However, CsYUC6 and CsYUC7 lack of the FAD-binding motif and GC motif (Supplemental Figure S3). Given that FAD-binding motif was shown to be essential for YUC monooxygenases activity^18^,^28^, CsYUC6 and CsYUC7 may function other than auxin biosynthesis in cucumber. Three cucumber YUC10 genes were identified, and CsYUC10s have several variants in the ATG-containing motif 1 and 2 which are highly conserved in FMO proteins (Fig. 2 and Supplemental Figure S3). In ATG-containing motif 2, CsYUC10b and CsYUC10c share the same divergence at the residue A which substituted by C (Supplemental Figure S3). This substitution has also been identified in the FvYUC10 in strawberry^24^. In addition, CsYUC10b owns a replacement at the site Y which was the same as strawberry FvYUC10 in the FMO-identifying sequence motif^16^,^24^, implying that YUC10 may evolve new function in the horticultural crops including cucumber and strawberry. CsYUC6, −8, −9 and −11 are highly conserved in all the FMO motifs, suggesting that the functions of these genes may be conserved in cucumber.

### Cucumber *CsYUCs* in response to abiotic stress

IAA, as the main endogenous auxin in plants, had two existing forms: free and conjugated^42^,^43^. Free IAA can be produced from *de novo* biosynthesis or released from IAA conjugates^11^,^43^. Under high temperature, free IAA level was significantly increased in cucumber seedlings (Fig. 4A). Correspondingly, *CsYUC8* and *CsYUC9* were selectively upregulated at 38 °C for 3 h (Fig. 4B), suggesting that in response to high temperature, cucumber plants elevate free IAA level to fight against heat injury by activating of *CsYUC8* and *CsYUC9*. In consistent with this finding, previous studies showed that *YUC8* and *YUC9* expression were upregulated by 29 °C treatment in *Arabidopsis* seedlings^44^, and that *YUC8* was activated by *PIF4* and thus endogenous IAA level was elevated during the high temperature-induced hypocotyl elongation in *Arabidopsis*^31^. Therefore, upregulation of *YUC8* and *YUC9* homologs may be a conserved mechanism to elevate endogenous IAA level in response to high temperature stress.

Despite auxin was shown to play important roles in various aspects of plant development^1^,^5^, the specific function of auxin under low temperature and salinity stress remains mysterious. The gravity response was inhibited under cold stress in *Arabidopsis* inflorescence, in which auxin may be involved^45^,^46^. Auxin-related genes including *Aux/IAA*, *GH3*, *ARF* were regulated by salinity stress in sorghum and *Arabidopsis*^47^,^48^. In this study, we found that endogenous IAA level were significantly increased and *CsYUC10b* transcripts were remarkably upregulated under low temperature and salinity stress (Fig. 4A,B). Further, *CsYUC4* was shown to antagonize *CsYUC10b* under low temperature, while *CsYUC10a* and *CsYUC11* act against *CsYUC10b* under salinity stress (Fig. 4B), suggesting that different *YUC* members participate in stress response, and even antagonize each other to create a buffering system for endogenous auxin synthesis in cucumber. Further functional analysis of 35S:CsYUC11 transgenic plants confirmed that *CsYUC11* enhanced plant tolerance to salt stress (Fig. 7A,E), but did not mediate response to freezing or heat stress (Fig. 7B–D).

### *CsYUC11* regulates pedicel elongation and stamen development in *Arabidopsis*

Several studies have shown the important roles of auxin in stem and pedicel elongation^49^,^50^. *CORYMBOSA1* (*CRM1*), a key regulator for pedicel elongation^50^, belongs to the membrane-associated protein family that functions in polar auxin transport and distribution^51^. Mutation in *CRM1* resulted in shorten pedicel in *Arabidopsis*^50^. Similarly, loss-of-function of *AUXIN RESISTANT 1* led to reduced auxin signaling and short pedicel in *Arabidopsis*^52^. On the other hand, overexpression of wheat *TaYUC10* in *Arabidopsis* produced elongated petiole^53^. Here we found that ectopic expression of cucumber *CsYUC11* displayed elongated pedicel in *Arabidopsis* (Fig. 6C–E), a typical phenotype for auxin over-accumulation, suggesting that *CsYUC11* regulates pedicel elongation through auxin biosynthesis. In addition, we found that *CsYUC11* is specifically expressed in the stamen of male flowers, and the pollens are defective and the fertility is reduced in the transgenic lines of 35S:CsYCU11 in *Arabidopsis* (Fig. 6F–I), supporting the essential function of *CsYUC11* in stamen development. However, *yuc2 yuc6* double mutants are sterile and *YUC6* is specifically expressed in stamens and pollens in *Arabidopsis*^15^. Overexpression of wheat *TaYUC10* resulted in pollen defects and sterility in *Arabidopsis*^53^. Therefore, stamen development is regulated by distinct *YUC* family genes in different species, and the specific role (positive or negative) of YUCs is species- and member- dependent. Further studies using the overexpression lines and knock-out lines of *CsYUCs* through genetic transformation in cucumber would shed light on the specific functions of *CsYUCs* during flower and fruit development.

## Materials and Methods

### Sequence database, gene structure construction, FMOs-domain identification and phylogenetic analysis

The sequence information of the 11 YUC proteins in *Arabidopsis* (AtYUCs) were obtained from TAIR (https://www.arabidopsis.org/index.jsp), followed by a BLAST search against the Cucumber Genome Database (http://cucumber.genomics.org.cn/page/cucumber/index.jsp) to obtain the sequence information of the 10 cucumber CsYUCs. The putative protein sequences were aligned using ClustalW and the Neighbor-Joining phylogenetic tree was constructed using the bootstrap analysis with 1000 replications in the MEGA5 software package^54^. The distribution of the CsYUCs on chromosomes was drawn manually based on the position information from the Cucumber Genome Database (Table S2). The gene structure analysis of *CsYUCs* was performed using the online tool GSDS (http://gsds.cbi.pku.edu.cn/). The conserved FMOs-domains were identified by the BoxShade web site (http://www.ch.embnet.org/software/BOX_form.html), and the repeats logos were obtained through the online tool (http://weblogo.berkeley.edu/logo.cgi)^55^.

### Plant material and growth conditions

Cucumber inbred line 1461 was used in this study, and grown in the Experimental Field of China Agricultural University at Beijing under standard greenhouse conditions. Water management and pest control were performed according to standard protocols. The *Arabidopsis thaliana Columbia* (*Col*) ecotypes and *35S:CsYUC11/Col* transgenic plants were grown in soil at 22 °C under 16 h/8 h light/dark condition in a growth chamber.

### Abiotic stress treatments and IAA measurement

The 21-day-old cucumber seedlings grown under 16 h/8 h and 25 °C/18 °C day/night condition were used for abiotic stress treatment. Seedlings were incubated at 4 °C or watering with 100 mM NaCl solution. For high temperature treatment, plants were kept in 16 h/8 h and 38 °C/28 °C. For shade treatment, plants were treated with or without simulated shade for 1 h, 3 h and 6 h. Each stress treatment was repeated three times. After stress treatment, cucumber shoot apices were harvested and immediately frozen in liquid nitrogen until further use. About 0.1 ~ 0.2 g samples were used for IAA measurement using enzyme-linked immunosorbent assay as described^56^. For abiotic treatments in the 35S:CsYUC11 transgenic *Arabidopsis,* WT and transgenic seeds were growing in Murashige/Skoog (MS) culture medium with 25 mg/L hygromycin for 4d, then the MS plate were replace by new MS plate containing 150 mM NaCl, seedling survival rate and root length were measured after cultured vertically for 3 days. For freezing treatment, the 3-week-old seedlings grown in soil were placed in a temperature chamber at −8 °C for 120 min. Survival rate of WT and transgenic plants were counted after resuming growth at 22 °C under 16 h/8 h light/dark condition for 7 days. For heat treatment, the 10-day-old seedlings grown in Murashige/Skoog (MS) culture medium with 25 mg/L hygromycin were heat treated at 45 °C for 2.5 hours, and survival rate were determined after resuming growth at 22 °C under 16 h/8 h light/dark condition for 5 days.

### Semiquantitative RT-PCR and quantitative real-time RT-PCR

Total RNA was extracted using Quick RNA isolation Kit (Waryoung, China). The first strand cDNAs were synthesized using TianScript II RT Kit (Tiangen, China). For semiquantitative RT-PCR, the primers and cycles were empirically optimized. Quantitative real-time RT-PCR was performed as described previously^57^. The cucumber *UBIQUITIN* and *Arabidopsis ACTIN2* genes were used as an internal control to normalize the expression data. Each qRT-PCR experiment was repeated three times. The primer information is listed in Supplemental Table S4.

### *In situ* hybridization

Cucumber male and female buds were fixed in 3.7% formol-acetic-alcohol (FAA), and *in situ* hybridization was performed as described^58^. The primer information is listed in Supplemental Table S4.

### Ectopic expression of *CsYUC11* in *Arabidopsis*

The full length coding sequence of *CsYUC11* was cloned into pCAMBIA1305.1 vector with 35S promoter through NcoI and SpeI sites. The construct was then introduced into Agrobacterium strain C58 by electroporation and transformed into *Arabidopsis* wide-type (*Col*) plants through the floral-dip method^59^. The transgenic plants were screened on MS medium with 25 mg/L hygromycin. The primer information is listed in Supplemental Table S4.

## Additional Information

**How to cite this article**: Yan, S. *et al.* Different cucumber *CsYUC* genes regulate response to abiotic stresses and flower development. *Sci. Rep.* **6**, 20760; doi: 10.1038/srep20760 (2016).

## Supplementary Material

Supplementary Information
