# Supplementary material for: Different cucumber CsYUC genes regulate response to abiotic stresses and flower development
Source: Sci Rep. 2016 Feb 9;6:20760. doi: 10.1038/srep20760 (PMC4746583; doi:10.1038/srep20760)
Supplement: Supplementary Information [file srep20760-s1.pdf]

Different cucumber *CsYUC* genes regulate response to abiotic stresses and flower development

Shuangshuang Yan, Gen Che, Lian Ding, Zijing Chen, Xiaofeng Liu, Hongyin Wang, Wensheng Zhao, Kang Ning, Jianyu Zhao, Kiflom Tesfamichael, Qian Wang[#], Xiaolan Zhang[#]

Figure S1

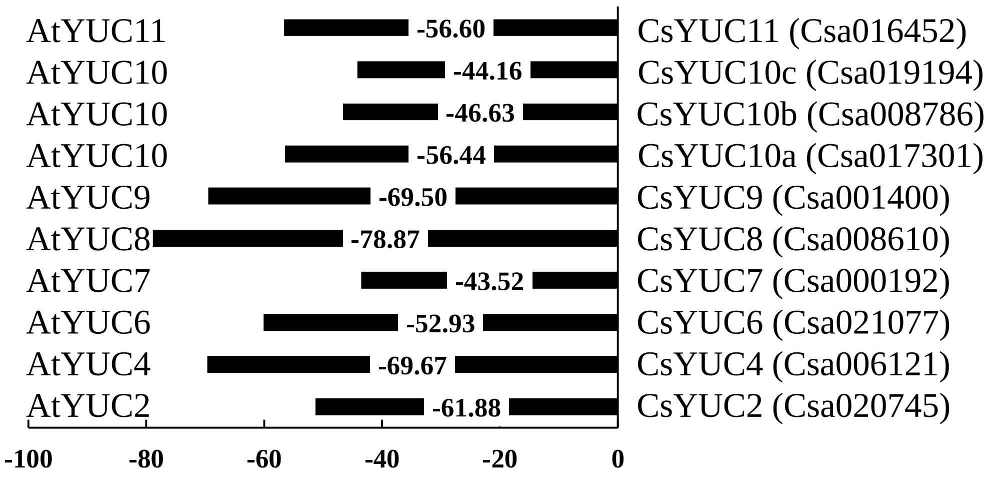

Supplemental Figure S1. Tornado chart showing the similarity of YUC proteins in cucumber and *Arabidopsis*.

Figure S2

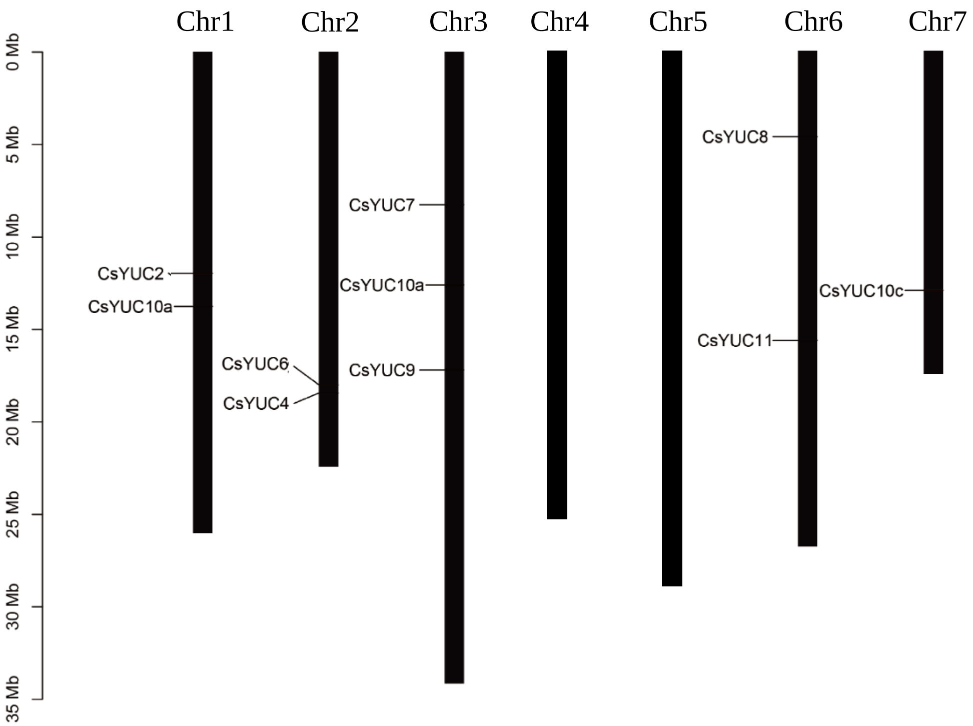

Supplemental Figure S2. **Distribution of the 10 *CsYUC* genes on cucumber chromosomes.**

The 10 *CsYUC* genes are localized on five chromosomes, and none was distributed on chromosome 4 and chromosome 5. The chromosome size is dimensioned by the left scale which is in megabases (Mb).

Figure S3

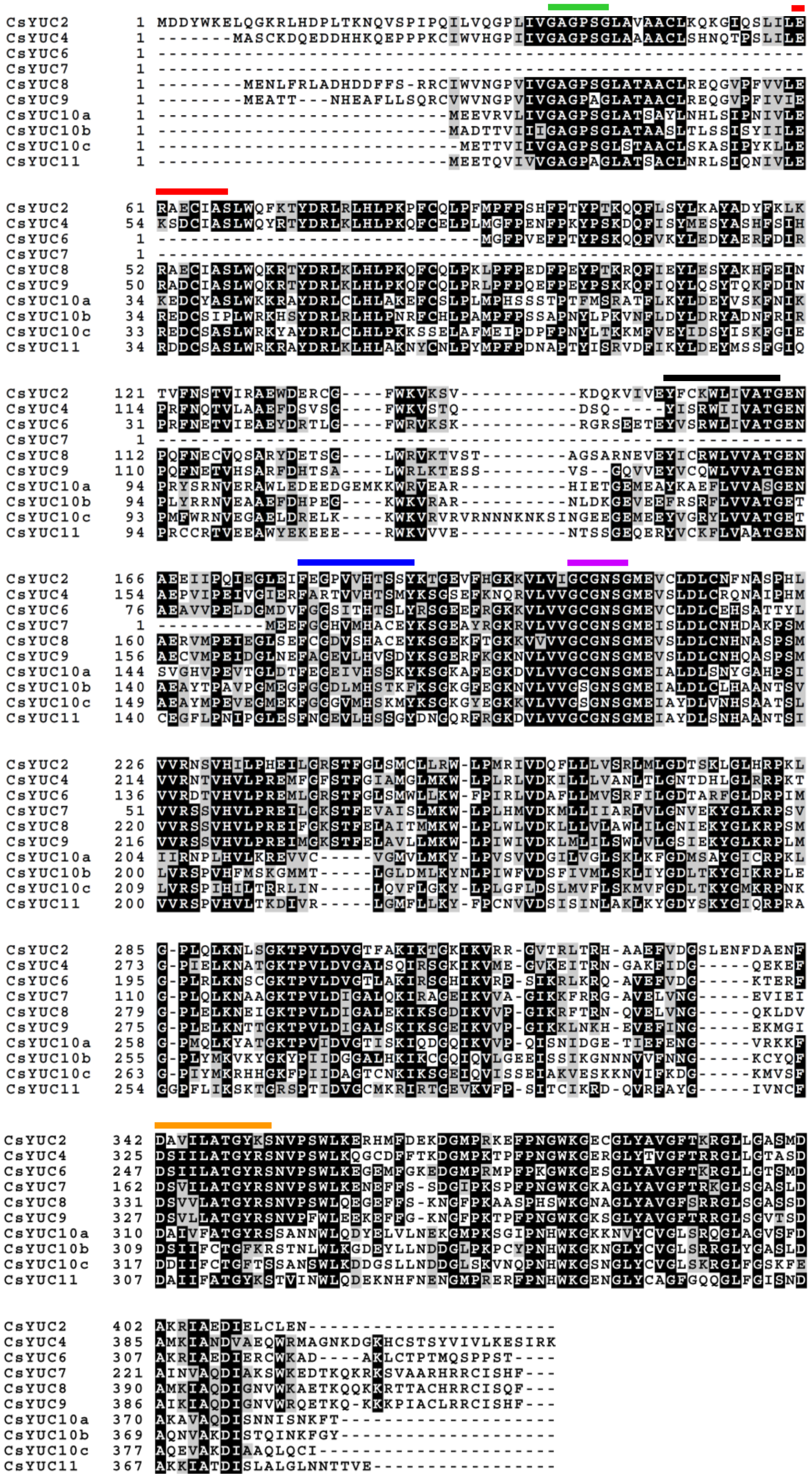

Supplemental Figure S3. Sequence alignment of the cucumber *YUC* family.
ClustalX alignment of the 10 YUC proteins in cucumber. Identical and similar amino acids are shaded in black and grey, respectively. The colored lines above the alignment represent relatively conserved flavin monooxygenase (FMO) domains. Green, FAD-binding motif; red, GC motif; black, ATG-containing motif 1; blue, FMO-identifying sequence; purple, NADPH-binding motif; orange, ATG-containing motif 2.

Figure S4

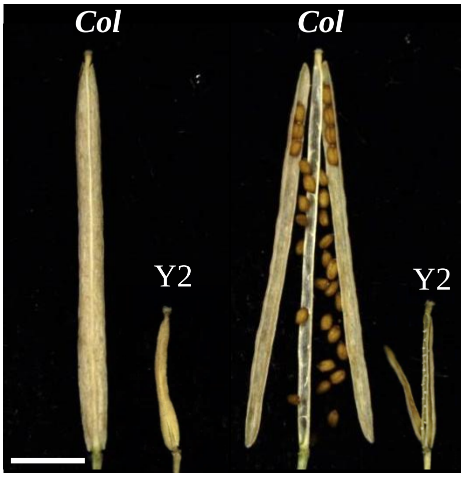

Supplemental Figure S4 Siliques ready for seed dispersal in WT(*Col*) and *CsYUC11* transgenic line Y2.

Table S1. Protein similarity between AtYUCs and CsYUCs

	AtYUC1	AtYUC2	AtYUC3	AtYUC4	AtYUC5	AtYUC6	AtYUC7	AtYUC8	AtYUC9	AtYUC10	AtYUC11
CsYUC2	50.00%	61.88%	49.21%	52.74%	48.86%	60.09%	50.57%	49.55%	49.31%	38.00%	40.42%
CsYUC4	65.57%	54.78%	52.83%	69.67%	51.98%	52.57%	52.29%	52.79%	52.93%	38.08%	41.22%
CsYUC6	44.15%	51.31%	42.47%	44.60%	43.06%	52.93%	42.96%	42.62%	43.26%	34.09%	35.56%
CsYUC7	31.88%	31.92%	42.11%	33.58%	40.33%	33.72%	43.52%	38.97%	40.62%	24.75%	25.55%
CsYUC8	53.30%	52.34%	68.42%	55.16%	76.71%	56.98%	69.44%	78.87%	74.59%	40.00%	43.43%
CsYUC9	52.38%	53.41%	63.39%	55.63%	67.92%	54.44%	64.12%	69.48%	69.50%	42.14%	43.94%
CsYUC10a	39.62%	40.57%	36.90%	40.28%	40.79%	37.73%	37.33%	39.63%	41.36%	56.44%	49.12%
CsYUC10b	39.34%	41.01%	40.14%	39.76%	37.70%	40.00%	38.30%	38.84%	40.14%	46.63%	42.68%
CsYUC10c	37.27%	39.81%	37.81%	37.67%	37.84%	40.00%	38.01%	36.70%	38.76%	44.16%	39.75%
CsYUC11	39.43%	40.87%	38.95%	42.00%	42.49%	39.86%	37.79%	39.95%	41.88%	50.39%	56.60%

Table S2. Information of exon number and gene length in *AtYUCs* and *CsYUCs*

	Exon Number	CDS Length(bp)	Exon Length(bp)				
			E1	E2	E3	E4	E5
AtYUC2	4	1248	680	249	122	197	
CsYUC2	4	1248	689	248	84	227	
AtYUC4	4	1236	627	249	121	239	
CsYUC4	4	1266	654	247	123	242	
AtYUC6	5	1281	666	246	124	179	66
CsYUC6	4	1008	420	247	123	218	
AtYUC7	3	1296	699	370	227		
CsYUC7	3	765	164	371	230		
AtYUC8	1	1281	1281				
CsYUC8	3	1272	671	370	231		
AtYUC9	2	1266	665	601			
CsYUC9	3	1257	659	371	227		
AtYUC10	4	1152	609	232	126	185	
CsYUC10a	5	1161	289	335	315	222	
CsYUC10b	4	1158	611	236	129	182	
CsYUC10c	4	1176	638	233	129	176	
AtYUC11	3	1176	616	357	203		
CsYUC11	4	1161	611	236	123	191	
AtYUC1	4	1245	645	248	123	229	
AtYUC3	3	1314	714	370	230		
AtYUC5	1	1275	1275				

Table S3. The accession number and genomic position of *CsYUCs* in cucumber

CsYUC	Accession number	Chromosome	Position	Chromosome (bp)
CsYUC2	Csa020745	Chr1	11957747:11960229	26004697
CsYUC4	Csa006121	Chr2	18431170:18433206	22393233
CsYUC6	Csa021077	Chr2	17999666:18001975	22393233
CsYUC7	Csa000192	Chr3	8253128:8254360	34138124
CsYUC8	Csa008610	Chr6	4627590:4629102	26767080
CsYUC9	Csa001400	Chr3	17187586:17189062	34138124
CsYUC10a	Csa017301	Chr2	13751577:13754260	22393233
CsYUC10b	Csa008786	Chr3	12587429:12590903	34138124
CsYUC10c	Csa019194	Chr7	12941281:12945616	17451012
CsYUC11	Csa016452	Chr6	15636916:15638418	26767080

Table S4. List of primer information used in this study

Gene	Forward primer (5'-3')	Reverse primer (5'-3')
**For gene amplification and vector construction**		
CsYUC11-cloning	ATGGAGGAAACGCAGGTGA	TCACTCCACAGTAGTGTTGTTTAGAC
CsYUC11-oe	CATGCCATGGATGGAGGAAACGCAGGTGA	GGACTAGTTCACTCCACAGTAGTGTTGTTTAGAC
**For transgenic plants identification**		
CaMV35S	GACGCACAATCCCACTATCC	
**For *in situ* hybridization**		
CsYUC11-T7	TGTAATACGACTCACTATAGGGTGCCCATTATCATAACCACTTG	
CsYUC11-Sp6	GATTTAGGTGACACTATAGAATGCTATGGAGGAAACGCAGGTGA	
**For qRT-PCR**		
CsYUC2	TGCAAGGCAAAAGACTTCACG	GCTATACATTCGGCTCTTTCAAGGA
CsYUC4	TGGCTAAAGCAGGGGTGTGA	CGTTGGCGATTTTCATAGCG
CsYUC6	GAAGTATTTGGAGGATTACGCTG	TGTTTCCTCAGAACGACCGC '
CsYUC7	GGTGAGGCTTACCGTGGGAAAC	CTTGGCATCATGGTTACAAAGA
CsYUC8	CATACGCCAAGCATTTTGAGAT	ATGTATTCGACCTCGTTACGGG
CsYUC9	CCGAGTCTTCCGTTTCTGGT	GGCATGACACACTCTGCATTTTC
CsYUC10a	GTCCTTCTGGCTTGGCTACCT	TGGCTAAGTGAAGGCATAAACG
CsYUC10b	CCTTCTGGTCTTGCCACTGC	CAAAACCGATTGGGGAGG
CsYUC10c	CTATCCACCGCCGCATGTTTA	CGCCAGCTCCGATGATTTCTT
CsYUC11	CAATGCACCGACGTATATTTCG	CCTCTCTTGCTCACCACTACTTGT
CsUBI-eq	CACCAAGCCCAAGAAGATC	TAAACCTAATCACCACCAGC
AtACTIN2	CCTTCGTCTTGATCTTGCGG	AGCGATGGCTGGAACAGAAC
AtYUC11	GGAAACACCAAAAATGTGGACTC	ACCATTCTTCCCCTTCCAGTG
